# Subfoveal Choroidal Thickness in Myopia: An OCT-Based Study in Young Chinese Patients

**DOI:** 10.1155/2020/5896016

**Published:** 2020-05-04

**Authors:** Fen Xiong, Jun Tu, Tian Mao, Li Yu, Nana Lin, Hongfei Liao

**Affiliations:** ^1^Affiliated Eye Hospital, Nanchang University, Nanchang 330006, China; ^2^Department of Orthopaedics, The First Affiliated Hospital of Nanchang University, Nanchang 330006, China

## Abstract

Myopia is a common cause of visual impairment worldwide. Choroidal thickness (ChT) reflects the characteristic changes in myopic children and may be used as an important index of myopia. The purpose of this study was to investigate ChT and its distribution across the posterior pole in young myopic Chinese patients using enhanced depth imaging optical coherence tomography (EDI-OCT) and to explore the factors associated with it. A total of 402 myopic Chinese patients aged 6–16 years who underwent complete ophthalmic examinations, including those for axial length, cycloplegic refraction, and intraocular pressure, were examined with EDI-OCT. The mean subfoveal ChT was 303.08 ± 76.87 *μ*m and displayed large variations at different positions (*p* < 0.05). The thickest sector was located 3 mm temporally from the fovea. Multivariate regression analysis showed a significant negative correlation of the subfoveal ChT values with axial length (AL), whereas the ChT was moderately influenced by the patient's sex. AL accounted for 7.9% of the ChT variance, whereas sex explained 9.6% of the ChT variance. In the population aged 11 years and older, AL accounted for 13.1% of the ChT variance. However, in those younger than 11 years, age was the only significant explanatory factor accounting for 5.2% of the ChT variance. In conclusion, we found a significant decrease in ChT with age in myopic children younger than 11 years. The negative association between age and ChT in children aged 11 years and older may be offset by the choroidal thickening mediated by pubertal growth spurts. The positive correlation between ChT and spherical equivalent in myopic adolescents aged 11 years and older suggests that the protective effect of lens thinning against rapid axial elongation disappears with age. Axial elongation becomes the dominant determinant of ChT in this age group.

## 1. Introduction

As a pigmented vascular tissue, the choroid serves a number of functions including the metabolic support of the retina [1]. In addition to its roles in nourishing the retina [2–6], the choroid plays an active role in changing the refractive state through the modulation of its thickness as demonstrated by Wallman et al. [7]. Furthermore, the choroid is uniquely situated to relay retina-derived signals to the sclera to effect changes in scleral extracellular matrix synthesis and ocular size, leading to refraction changes [8]. Thus, the choroid may play an important role in the etiology of myopia [7, 8].

Enhanced depth imaging optical coherence tomography (EDI-OCT) [9] enables in vivo cross-sectional visualization of the choroid and has allowed a better understanding of choroidal changes [10–12]. As a non-invasive, secure, economic, and convenient real-time imaging technique [13], it is widely used in ophthalmology.

The most common application of EDI-OCT in the analysis of refractive error is the detection of significantly decreased choroidal thickness (ChT) in patients with myopia [4, 14–16] in both the stromal and vascular components [17]. ChT has been associated with age [4, 15, 16, 18], refractive error [7, 17, 19–23], axial length (AL) [15, 16, 18, 20–22, 24–26], and sex [16, 18, 23–26], in both adults and children with conflicting results.

Myopia is one of the most common causes of visual impairment worldwide. Since children aged 6 to 16 years show the greatest prevalence in myopia, we chose to investigate ChT in this group of patients.

ChT increases significantly from early childhood to adolescence in emmetropes [23, 25, 26]. Another study on growth patterns in ocular biometry reported that ChT was positively associated with increasing age only in emmetropes and hyperopes, but negatively associated with age in myopes, with borderline significance (*p*=0.051) [15]. Consequently, we aimed to examine the thickness of the choroid and evaluate its variability across the posterior pole in a myopic pediatric population, stratified by age.

## 2. Patients, Materials, and Methods

The study was conducted in the Nanchang University Affiliated Eye Hospital, and all participants were recruited from the Ophthalmology Outpatient Clinic and examined in the Pediatric Ophthalmology Department. The study was performed in adherence to the tenets of the *Declaration of Helsinki*. All parents declared that their children were healthy. All children and their parents were informed about the study procedure, and signed informed consent was obtained for all the guardians of the participants. Ethical approval for the study was obtained from the Nanchang University Clinical Research Centre.

A total of 402 myopic Chinese patients with varying degrees of myopia were recruited from November 2017 to March 2019. The inclusion criteria were age between 6 and 16 years, spherical power plus half cylinder power −6.00 D < spherical equivalent refraction (SER) < −0.50 D (cycloplegic refraction by cyclopentanone), and non-contact intraocular (NCT) pressure 10–21 mmHg. The exclusion criteria were presence of pathological myopia, amblyopia, and strabismus; previous history of ocular disease or surgery; and poor-quality OCT images. The research team consisted of one ophthalmologist, one optometrist, and three nurses.

Each enrolled patient underwent a complete ophthalmologic examination including monocular best corrected visual acuity testing using linear logarithm of the minimum angle of resolution (logMAR) charts, intraocular pressure (IOP) measurement by non-contact tonometry (model NT-4000, Nidek Inc., Fremont, CA, USA), slit-lamp examination (Haag-Streit slit-lamp, Köniz, Switzerland), dilated fundus examination with direct ophthalmoscopy, cycloplegic refraction, and axial length measurement. Pupillary dilation was induced by the instillation of three drops of 1% cyclopentolate in each eye (Cyclogyl; Alcon, Fort Worth, TX, USA) at 10-minute intervals, following which the pupil size and light reflex were examined. Cycloplegia was deemed complete if the pupil had dilated to ≥6 mm and light reflex was absent. The autorefractometer (ARK-700A, Nidek, Japan) was set to generate five valid refraction readings and the median value recorded by the instrument was used for analysis. SER was obtained with the following formula: SER = *S* + *C*/2. Axial length was measured with the IOL Master (Carl Zeiss Meditec Inc, Dublin, CA).

**Figure 1:**
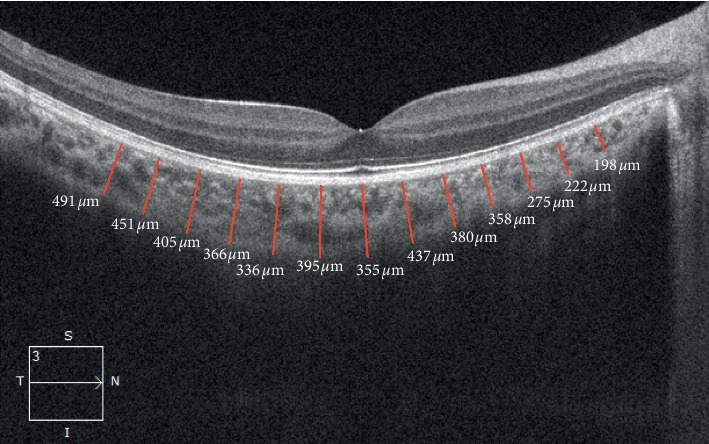
An example of measurement of choroidal thickness along the center line (Cf). Choroidal thickness was measured from the retinal pigment epithelium to the choroidal-scleral junction at 0.5 mm intervals.

Following the initial screening examination, each child underwent spectral domain EDI-OCT for chorioretinal imaging of his/her right eye with the Cirrus Spectral Domain OCT (Carl Zeiss Meditec Inc., Dublin, CA, USA). Images with signal strength <9 were excluded. Black and white EDI-OCT images were examined manually by measuring the ChT using the specific cursor provided by the machine. Thirteen points, including the central point of the centerline were chosen in 0.5-mm intervals to calculate the ChT (Figure 1). The images in which the choroidal borders could not be clearly detected were excluded from the analysis. All patients underwent EDI-OCT choroidal imaging in the morning clinic between 8 AM and 2 PM to avoid the variations in temporal ChT that may occur during the day [27, 28].

## 3. Statistical Analyses

Statistical analyses were performed using IBM SPSS statistics version 20.0 (IBM Co., Armonk, NY, USA). Mean, standard deviation (SD), and median were calculated for continuous variables. Comparisons between sex and age groups were performed using two-tailed Student's *t*-tests. Repeated measures analysis of variance was used to compare the ChT values at different points. Greenhouse-Geisser corrections were used for analyzing values that did not meet the Mauchly's test of sphericity. The associated factors for ChT in different age groups were analyzed by multivariate regression analysis, and *p*-values <0.05 were considered significant. To consider the relationship between the variables, this study incorporated linear as well as non-linear models.

## 4. Results

A total of 402 myopes (190 boys and 212 girls) were enrolled in this study. The mean age was 10.97 ± 2.24 years (range 6–16), mean axial length was 24.67 ± 0.93 mm (range 22.25–27.27), mean IOP was 15.61 ± 2.55 mmHg (range 10–21 mmHg), and the mean SER was −2.68 ± 1.41 D (range −0.50 to −6.00 D). The general characteristics of the participants are listed in Table 1. There were no significant differences between boys and girls for mean age, SER, IOP, or ChT (two-tailed Student's *t*-tests: *p*=0.69, 0.16, 0.952, and 0.376, respectively), whereas the mean axial length was significantly larger in boys than in girls (*p* < 0.01; Table 1).

**Figure 2:**
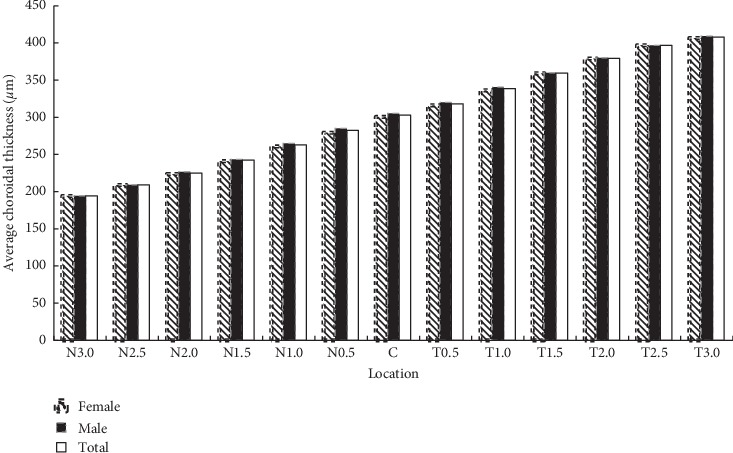
Choroidal thickness in micrometers versus location in myopic patients. The choroid was the thickest at the point 3 mm temporal to the fovea and decreased nasally to a minimum at the point 3 mm nasal to the fovea.

ChT displayed large variations among different points (*F* = 196.488; *p* < 0.001). The point located 3 mm temporally from the fovea had the thickest choroid with a mean thickness of 407.93 ± 91.65 *μ*m, followed by the central fovea sector. ChT decreased gradually toward the nasal sector horizontally. As illustrated in Table 2 and Figure 2, ChT did not exhibit significant differences by sex at any of the points (all *p* > 0.05).

**Figure 3:**
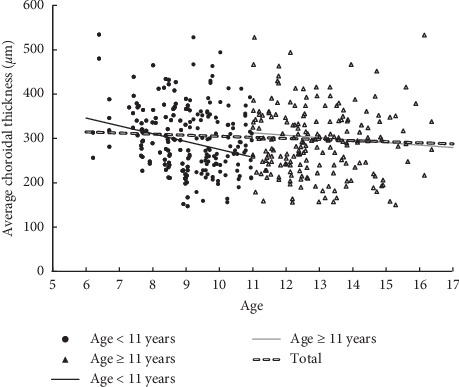
Scatterplot showing the correlation of subfoveal choroidal thickness versus age.

There were no significant differences between age groups in the general characteristics of the participants, except that children aged ≥11 years showed longer axial length (Table 3). We observed a trend for negative correlation between age and ChT (Table 4; Figure 3), but statistical significance was only observed in children <11 years.

**Figure 4:**
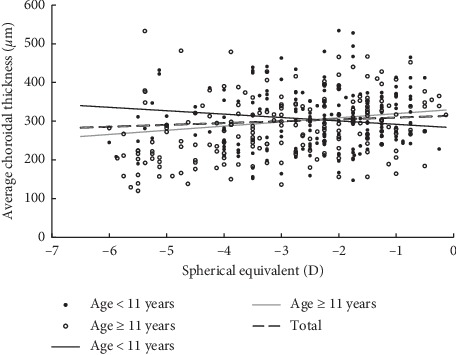
Scatterplot showing the correlation of subfoveal choroidal thickness with respect to spherical equivalent.

We found no significant relationship between SER and ChT in either population, i.e., those aged ≥11 years or those aged <11 years (Table 4; Figure 4). Furthermore, after classifying the data by refractive status, no significant relationships were observed between SER and ChT in the low (−3.00 D ≤ SER ≤ −0.50 D), moderate (−3.00 D < SER ≤ −5.00 D), or high (SER < −5.00 D) myopia groups (*p* > 0.05). However, individuals with high myopia had significantly thinner ChT than those with low or moderate myopia, although statistical significance was only observed in the temporal sectors (*p*-values: *p*_N3.0_=0.277, *p*_N2.5_=0.157, *p*_N2.0_=0.245, *p*_N1.5_=0.166, *p*_N1.0_=0.297, *p*_N0.5_=0.330, *p*_Foveal_=0.258, *p*_T0.5_=0.045, *p*_T1.0_=0.044, *p*_T1.5_=0.027, *p*_T2.0_=0.022, *p*_T2.5_=0.017, and *p*_T3.0_=0.021).

**Figure 5:**
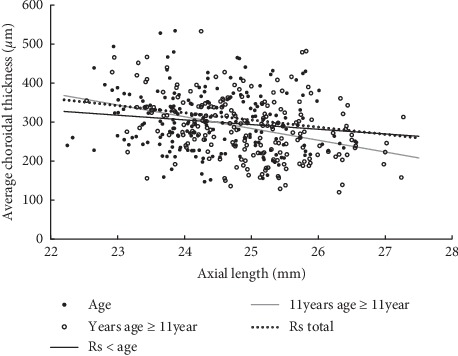
Scatterplot showing the correlation of subfoveal choroidal thickness (ChT) versus axial length. Children aged *l* < 11 years displayed a slower decrease in subfoveal ChT than children aged ≥11 years per mm of increase in axial length.

The mean ChT of all 13 horizontal lines except T3.0, correlated with the axial length significantly and inversely. The central foveal ChT displayed the fastest decrease, described by the formula: ChT = 755.305–18.70 ∗ AL mm (*p*=0.001; Figure 5). The mean central foveal ChT decreased with axial length (*p*=0.001; Table 4). Additionally, children younger than 11 years displayed a slower decrease of subfoveal ChT per mm increase in the axial length (Table 4).

Multiple linear regression showed a significant negative correlation of the subfoveal ChT values with axial length and a moderately significant effect of sex was observed in the multivariate model. In this model, AL accounted for 7.9% of the ChT variance, whereas sex accounted for 9.6% of the variance. The multiple regression formula used was central foveal ChT = 906.52–26.08 ∗ AL mm + 21.10 ∗ sex (female = 1, male = 2; *p* < 0.01). In the study population aged 11 years or older, AL accounted for 13.1% of the ChT variance. The multiple linear regression formula used was central foveal ChT = 1052.90–30.78 ∗ AL mm (*p* < 0.001). In the population <11 years, only age showed significant correlation with ChT accounting for 5.2% of its variance. The multiple regression formula used was central foveal ChT = 444.85 − 15.70 ∗ age years (*p* < 0.001). The multiple nonlinear regression formula used was ChT = 534.44 + 5.23 ∗ Age^2^ − 158.17 ∗ Age − 1.65 ∗ AL^2^ + 52.74 ∗ AL + 2.09 ∗ Age ∗ AL for age <11 years, while ChT = 5719.89 − 1.50 ∗ Age^2^ − 27.29 ∗ Age + 6.53 ∗ AL^2^ − 389.65 ∗ AL + 2.55 ∗ Age ∗ AL for age ≥11 years. Compared with linear regression, *R*^2^ only evaluated to 0.08 for age <11 years while *R*^2^ only evaluated to 0.15 for age ≥11 years.

## 5. Discussion

Multiple studies reported a ChT of 251–341 *μ*m in children with a mean SER of ±0.05 D, and 309.57–354 *μ*m in healthy adults of different races [22, 25, 26, 29–31]. However, the ChT of myopic children aged 10–15 years was reported to be 303 *μ*m [16, 23]. In our study, the mean subfoveal ChT in the myopic children aged 6–16 years was 294.16 ± 77.59 *μ*m. Although the values differed to varying degrees possibly because of differences in the instruments used, age, AL, race, and distribution of the refractive error among the individuals recruited in the various studies, the ChT ranged between 250 and 300 *μ*m in agreement with our findings.

The mean ChT measured in our study was between the thickest temporal and the thinnest nasal values in consensus with previously published studies [15]. However, according to Ikuno et al. [22] and Park et al. [31], the central point showed the thickest ChT in the adult population. Furthermore, ChT exhibited significant variation across the posterior pole in healthy children, with it being thicker in the central regions [25]. In consensus, we found the choroid to be thickest in the central point in some children although it was not statistically significant. The discordance between these studies might be due to the differences in the distribution of age and refractive error in the recruited individuals. We also found that the central foveal choroid displayed the fastest decrease with increasing AL when compared with the temporal or nasal choroid, in agreement with that of the temporal location which exhibited the smallest change with time [16]. Furthermore, the differences between myopic children in our study and emmetropes indicate that myopia may lead to much larger reductions in the subfovea than in the temporal choroid [25].

The role of age in influencing ChT has been controversial. Previous studies have shown that increasing age was associated with decreased ChT in the adult population. This relationship was quantified as a decrease in subfoveal ChT by 1.4–2.67 *μ*m per year of life [22, 31–33]. However, ChT has been confirmed to increase significantly from early childhood to adolescence in emmetropes [23, 25, 26]. These findings follow the developmental pattern reported in school-aged children exhibiting ChT thickening in an 18-month follow-up period, as shown by Read et al. [16]. This is because of the fact that these study participants featured a higher proportion of emmetropic and hyperopic children. Another study on ocular biometry reported a positive correlation between choroid thickness and age in emmetropes and hyperopes, and a negative correlation in myopes with borderline significance (*p*=0.051) [15]. In our analysis we found a similar negative correlation between age and ChT in children aged 6–16 years, but without statistical significance (*p*=0.11).

In a cross-sectional, observational study of 11–12-year-old children, ChT was observed to increase with the onset of pubertal growth. We found a negative association between age and subfoveal ChT only in children younger than 11 years. Thus, we consider 11 years to be the age below which the choroid thickness shows negative correlation with age in myopic children. As discussed above, the discordance between the age groups may be due to the beginning of puberty. Specifically, the negative association between age and ChT in children aged ≥11 years may be offset by choroidal thickening, which is mediated by pubertal growth [34]. Therefore, choroidal thickening may reflect normal growth of the vascular and connective tissue within the choroid or alternatively, could represent changes in blood flow with age. Although changes in ocular blood flow with age have been documented in adults, to our best knowledge, this phenomenon has not yet been examined in early childhood [25, 35, 36].

A positive correlation between ChT and SER refraction has been well established in previous studies in both children and adults [17, 21–23]. We observed a 10.76 *μ*m decrease in subfoveal ChT for each diopter of myopic shift only in children aged ≥11 years with borderline statistical significance (*p*=0.08), but not in children <11 years. The lens decreases its thickness and power until the age of 10 [37]. This decrease in lens power reduces the myopic shifts that normally would be associated with increases in AL showing that the lens play a key role in refractive development [20]. The positive correlation between SER and ChT in myopes aged ≥11 years might indicate the disappearance of the protective effect of lens thinning against rapid axial elongation typically associated with the progression of myopia.

AL has been proposed as the biometric parameter that most strongly correlates with SER and it plays a crucial role in the subfoveal ChT. The observed −18.46 *μ*m/mm effect of AL on subfoveal ChT was in agreement with previous studies that reported a decrease in subfoveal ChT by 16–58.2 *μ*m per mm increase in axial length in both children and adults [15, 16, 18, 21, 22, 24–26]. Read et al. assessed ChT in school-aged children and found that those undergoing faster axial eye growth exhibited less thickening while a few cases of choroidal thinning were observed [16]. The current study found that children <11 years displayed a slower decrease in subfoveal ChT per mm of AL increase (Figure 5; Table 4). Age may contribute to a slower reduction in children <11 years. In our multivariate regression analysis that was limited to the population < 11 years, only age showed significant correlation with ChT and accounted for 5.2% of its variance.

The relationship between sex and ChT in children has been the subject of many controversies. Our assessment of choroidal thickness after adjusting for other factors indicated that ChT was greater in boys than in girls, which was in contrast to the results of Li et al. [34]. In our multivariate regression analysis, AL accounted for 7.9% of the ChT variability, whereas sex accounted for 9.6% of the variability. In Li et al.'s study, ChT was lesser in boys and it increased with height and the onset of puberty in girls. This discordance between the results might be due to the different age distribution in the individuals recruited. The study population of Xiao et al. had a higher proportion of pubertal children, and girls may have attained puberty earlier than boys did. The difference in ethnicity of the participants between these studies is another possible explanation for the discrepancy.

IOP showed no significant association with ChT in our study. Although some studies have indicated IOP changes as a potential mechanism in the thermoregulation of the retina by the choroid [1, 38], other studies showed no such relationship [30, 39] and the significance of these observations remain unclear.

Our study has some limitations. First, our study design makes it difficult to attribute causation or to understand the time course of the various changes observed. A natural future perspective for our research is to investigate the role of choroid in the development of myopia in longitudinal studies, which will help clarify the temporal changes in the choroid. Second, previous studies have demonstrated a positive correlation between ChT and SER [17, 21–23], suggesting a potentially important role of the choroid in the development of refractive errors. However, we only found such an effect in the population aged ≥11 years with borderline significance, suggesting that the protective effect of the lens thinning with age against rapid axial elongation disappears with age and that axial elongation becomes the dominant determinant of ChT around the age of 11 years. Therefore, future studies among a larger population of children <11 years are needed to determine the choroidal growth profile of this age group.

## 6. Conclusions

We examined the ChT of a relatively large population of myopic children. We found a significant decrease in ChT with age in myopic children <11 years, which then appears to be affected by the pubertal growth spurt. Furthermore, we found a positive correlation between ChT and SER in the myopic population aged ≥11 years with borderline significance. This suggests that the protective effect of the lens thinning against rapid axial elongation disappears with age, whereas axial elongation becomes the dominant determinant of ChT around the age of 11 years. We should monitor the change of ChT in the children with increase in AL though the SER stained unchange to avoid fundus lesion induced by the thinner of choroid in myopic children ≥11. Future longitudinal studies will help clarify the temporal course of change in ChT and the potential influence of these changes on the development of refractive error in childhood.

## Figures and Tables

**Table 1:** Demographic information of 402 myopic children.

	Total, *n* = 402	Boys, *n* = 190	Girls, *n* = 212	*p*
Age, y	10.97 ± 2.24	11.02 ± 2.25	10.93 ± 2.23	0.689
Axial length, mm	24.67 ± 0.93	24.98 ± 0.91	24.40 ± 0.87	**<0.05**
Spherical equivalent refractive error, diopter	−2.68 ± 1.41	−2.78 ± 1.41	−2.58 ± 1.40	0.164
Intraocular pressure, mmHg	15.61 ± 2.55	15.63 ± 2.38	15.60 ± 2.71	0.952
Choroidal thickness, *μ*m	294.16 ± 77.59	297.80 ± 80.62	290.90 ± 74.82	0.376

Data are shown as mean ± standard deviation; *p*-values in boldface text indicate statistical significance.

**Table 2:** Mean choroidal thickness of all patients, Mean ± SD, *μ*m.

	Total, *n* = 222	Boys, *n* = 112	Girls, *n* = 110	*p*
N3.0	194.2 ± 61.73	194.31 ± 63.11	194.09 ± 60.57	0.97
N2.5	209.06 ± 64.68	209.29 ± 64.87	208.84 ± 64.79	0.95
N2.0	225.17 ± 67.42	226.56 ± 69.59	223.75 ± 65.44	0.75
N1.5	242.27 ± 67.6	243.45 ± 70.92	241.07 ± 64.34	0.79
N1.0	262.86 ± 71.17	264.79 ± 74.81	260.89 ± 67.54	0.68
N0.5	282.35 ± 75.81	285.26 ± 80.73	279.39 ± 70.71	0.56
C	303.08 ± 76.87	305.5 ± 82.35	300.61 ± 71.14	0.63
T0.5	318.16 ± 79.67	320.01 ± 84.8	316.28 ± 74.43	0.72
T1.0	338.53 ± 80.59	340.66 ± 85.15	336.36 ± 75.98	0.69
T1.5	359.62 ± 83.16	359.83 ± 85.36	359.4 ± 81.26	0.96
T2.0	379.53 ± 85.16	380.03 ± 86.8	379.03 ± 83.86	0.93
T2.5	396.75 ± 87.11	396.6 ± 88.11	396.9 ± 86.48	0.97
T3.0	407.93 ± 91.65	409.21 ± 91.03	406.63 ± 92.67	0.83

N3.0, N2.5, N 2.0, N1.5 N1.0, N0.5: points 3.0, 2.5, 2.0, 1.5, 1.0, and 0.5 mm nasal to the central point of the horizontal line. C: central point of the horizontal line. T3.0, T2.5, *T* 2.0, T1.5, T1.0, T0.5: points 3.0, 2.5, 2.0, 1.5, 1.0, and 0.5 mm temporal to the central point of the horizontal line. Data are shown as mean ± standard deviation.

**Table 3:** Comparison between age groups.

	Total (*n* = 402)	Age < 11 (*n* = 205)	Age ≥ 11 (*n* = 197)	*p*
Age, y	10.97 ± 2.24	9.12 ± 1.1	12.9 ± 1.3	<0.001
Axial length, mm	24.67 ± 0.93	24.43 ± 0.87	24.92 ± 0.93	**<0.05**
Spherical equivalent refractive error, diopter	−2.68 ± 1.41	−2.41 ± 1.23	−2.95 ± 1.53	0.12
Intraocular pressure, mmHg	15.61 ± 2.55	15.54 ± 2.47	15.68 ± 2.70	0.97
Choroidal thickness, *μ*m	294.16 ± 77.59	302.2 ± 75.15	285.8 ± 79.38	0.459

Data are shown as mean ± standard deviation; *p*-values in boldface indicate statistical significance.

**Table 4:** Factors associated with central foveal choroid in myopic children.

Variables	Age < 11 (*n* = 205)		Age ≥ 11 (*n* = 197)		Total	
	Estimate (95% CI)	*p*	Estimate (95% CI)	*p*	Estimate (95% CI)	*p*
Age	−15.70 (−24.884 ∼ 6.512)	<0.001	−5.54 (−15.468 ∼ 4.384)	0.49	−2.75 (−6.641 ∼ 1.761)	0.11
Spherical equivalent refractive error	−8.78 (−21.752 ∼ 4.190)	0.18	10.76 (−2.157 ∼ 19.357)	0.08	4.74 (−2.408 ∼ 11.889)	0.19
Axial length	−12.03 (−29.283 ∼ 5.229)	0.17	−30.18 (−40.87 ∼ 19.47)	<0.001	−18.46 (−28.874 ∼ −8.021)	<0.001
